# Environmental Antimicrobial Resistance: Sources, Dissemination, Risks, and Mitigation Strategies Within a One Health Framework

**DOI:** 10.3390/antibiotics15090849

**Published:** 2026-08-31

**Authors:** Takashi Azuma

**Affiliations:** 1 Department of Pharmacy, Osaka Medical and Pharmaceutical University, Takatsuki 569-1094, Japan; takashi.azuma@ompu.ac.jp or t.azuma.mail@gmail.com; Tel./Fax: +81-72-690-1055; 2 Center for Infectious Disease Education and Research (CiDER), The University of Osaka, Suita 565-0871, Japan

Antimicrobial resistance (AMR) has emerged as one of the most pressing global public health challenges of the twenty-first century [1]. Although the clinical consequences of AMR have long been recognized, increasing attention is now being directed toward the environment as a critical reservoir, dissemination pathway, and potential driver of the persistence and evolution of antimicrobial resistance. Environmental compartments, including wastewater, surface waters, soils, sediments, and agricultural ecosystems, receive continuous inputs of antimicrobial compounds, antimicrobial-resistant bacteria (ARB), antimicrobial resistance genes (ARGs), and mobile genetic elements originating from human, veterinary, agricultural, industrial, and other anthropogenic activities. These interconnected environmental reservoirs may facilitate the persistence, selection, and dissemination of resistance determinants, thereby reinforcing the importance of environmental AMR within the One Health framework, which recognizes the interdependence of human, animal, and environmental health [2].

The Special Issue entitled Environmental Antimicrobial Resistance: Sources, Dissemination, Risks, and Mitigation Strategies was established to advance the understanding of the occurrence, fate, dissemination, ecological and public health implications, and mitigation of AMR in environmental settings. The Special Issue provided a multidisciplinary platform for research addressing environmental reservoirs of resistance, mechanisms and pathways of dissemination, surveillance strategies, risk assessment, and emerging approaches for AMR mitigation. The breadth of topics represented in this collection reflects the increasingly interdisciplinary nature of environmental AMR research and its growing relevance to researchers, public health professionals, environmental engineers, healthcare practitioners, policymakers, and other stakeholders.

The contributions to this Special Issue collectively highlight several interconnected themes. First, they reinforce the concept that environmental matrices should not be regarded merely as passive recipients of antimicrobial contaminants and resistant microorganisms, but rather as dynamic and interconnected components of the broader AMR landscape. Wastewater systems, hospital effluents, municipal wastewater treatment plants, agricultural environments, and receiving waters can act as convergence points for antimicrobial residues, resistant microorganisms, and genetic determinants of resistance. Their interactions with physicochemical and biological conditions may influence the persistence, selection, and dissemination of resistance [3]. Understanding these processes is therefore essential for identifying environmental hotspots and potential intervention points along transmission pathways.

Second, the contributions emphasize the importance of integrated and multidisciplinary surveillance. Advances in molecular microbiology, next-generation sequencing, metagenomics, and quantitative analytical chemistry have substantially expanded the capacity to characterize environmental resistomes, detect resistance determinants, and monitor antimicrobial contamination. Integrating microbiological, molecular, chemical, and environmental information can provide a more comprehensive characterization of AMR than any individual approach alone. Such integration is particularly important for distinguishing the presence of resistance determinants from their potential environmental significance and for establishing evidence-based surveillance frameworks [4].

Third, the Special Issue highlights the continuing challenges associated with environmental AMR risk assessment. Although the occurrence of antimicrobial compounds, ARB, and ARGs in environmental matrices is increasingly documented, translating occurrence data into quantitative and biologically meaningful estimates of ecological and public health risk remains difficult. Important uncertainties persist regarding exposure assessment, resistance selection, persistence and mobility of resistance determinants, horizontal gene transfer, and the links between environmental resistance and clinically relevant outcomes. Accordingly, improved approaches for integrating chemical, microbiological, molecular, and epidemiological evidence will be essential for advancing environmental AMR risk assessment and supporting risk-informed decision-making [5].

A fourth major theme concerns mitigation and intervention strategies. Effective control of environmental AMR requires measures that extend beyond conventional clinical antimicrobial stewardship. Source control, improved wastewater treatment, targeted removal of antimicrobial residues and resistant microorganisms, environmental surveillance, and policies aimed at reducing the release of antimicrobial compounds and resistance determinants may all contribute to reducing environmental AMR burdens. At the same time, the effectiveness of individual interventions should be evaluated not only in terms of contaminant removal, but also with respect to their potential effects on resistance selection, dissemination, and overall environmental risk [6]. This broader perspective is important because reductions in chemical concentrations or bacterial abundance do not necessarily equate to proportional reductions in AMR risk.

Importantly, the collection underscores the necessity of a One Health perspective for addressing environmental AMR. Resistance does not respect disciplinary, environmental, or geographical boundaries. Human activities can shape environmental reservoirs of resistance, while environmental pathways may in turn contribute to the circulation of resistant microorganisms and resistance determinants relevant to human and animal health. Consequently, meaningful progress requires collaboration across microbiology, environmental science, analytical chemistry, epidemiology, clinical medicine, veterinary medicine, engineering, public health, and policy. The integration of these perspectives is essential for moving from fragmented surveillance toward a systems-level understanding of AMR and for identifying intervention strategies that can deliver benefits across multiple sectors [7].

Despite substantial progress in the field, important knowledge gaps remain. Future research should strengthen the evidence linking environmental AMR exposures with clinically and ecologically relevant outcomes, improve quantitative and mechanistic approaches to risk assessment, establish harmonized and comparable surveillance methodologies, and evaluate the long-term effectiveness and unintended consequences of mitigation technologies. Greater attention should also be given to the connectivity among environmental compartments and to the identification of critical control points along the continuum from antimicrobial use and release to environmental persistence, resistance selection, dissemination, and eventual human or animal exposure. In parallel, greater integration of chemical monitoring, culture-based microbiology, molecular surveillance, and genomic approaches will be important for developing a more comprehensive and actionable understanding of environmental AMR.

As Guest Editor, I would like to express my sincere appreciation to all authors who contributed their valuable research to this Special Issue. I am also deeply grateful to the reviewers for their expertise, time, and constructive comments, which were instrumental in maintaining the scientific quality of the published contributions. Further, I would like to thank the editorial team of Antibiotics for their professional assistance and continued support throughout the development and publication of this Special Issue.

The successful completion of this Special Issue and its subsequent publication as a reprint volume reflect the growing scientific interest in environmental AMR and the importance of addressing resistance beyond the clinical setting. I hope that the research collected in this volume will stimulate further interdisciplinary investigation, promote closer integration of environmental and public health perspectives, and contribute to the development of scientifically robust and practically implementable strategies for mitigating AMR within a One Health framework.
